# Sepsis-related deaths in Brazil: an analysis of the national mortality registry from 2002 to 2010

**DOI:** 10.1186/s13054-014-0608-8

**Published:** 2014-11-05

**Authors:** Leandro U Taniguchi, Ana Luiza Bierrenbach, Cristiana M Toscano, Guilherme PP Schettino, Luciano CP Azevedo

**Affiliations:** Research and Education Institute (IEP), Hospital Sirio-Libanes, Rua Cel, Nicolau dos Santos 69, São Paulo, Brazil; Emergency Medicine Discipline, Hospital das Clínicas da Faculdade de Medicina da Universidade de São Paulo, Av Enéas de Carvalho Aguiar 255 Sala 5023, São Paulo, Brazil; Sanas Epidemiology and Research, Avenida Paulista 2073, Edifício Horsa 1, salas 703/704, São Paulo, Brazil; Department of Collective Health, Federal University of Goias, Rua 235 s/n, Goias, Brazil

## Abstract

**Introduction:**

Limited population-based epidemiologic information about sepsis’ demography, including its mortality and temporal changes is available from developing countries. We investigated the epidemiology of sepsis deaths in Brazil using secondary data from the Brazilian Mortality Information System.

**Methods:**

Retrospective descriptive analysis of Brazilian multiple-cause-of-death data between 2002 and 2010, with sepsis-associated International Classification of Diseases, 10th Revision (ICD-10) code indicated as the cause of death. Population-based sepsis associated mortality rates and trends were estimated. Annual population-based mortality rates were calculated using age-stratified population estimates from the 2010 census provided by the Brazilian Institute of Geography and Statistics as denominators.

**Results:**

The total number of annual deaths recorded in Brazil increased over the decade, from 982,294 deaths reported in 2002 to 1,133,761 deaths reported in 2010. The number of sepsis associated deaths also increased both in absolute numbers and proportions from 95,972 (9.77% of total deaths) in 2002 to 186,712 deaths (16.46%) in 2010. The age-adjusted rate of sepsis-associated mortality increased from 69.5 deaths per 100,000 to 97.8 deaths per 100,000 population from 2002 to 2010 (*P* <0.001). Sepsis-associated mortality was higher in individuals older than 60 years of age as compared to subjects aged 0 to 20 years (adjusted rate ratio 15.7 (95% confidence interval (CI) 15.6 to 15.8)) and in male subjects (1.15 (95% CI 1.15 to 1.16)).

**Conclusions:**

Between 2002 and 2010 the contribution of sepsis to all cause mortality as reported in multiple-cause-of-death forms increased significantly in Brazil. Age-adjusted mortality rates by sepsis also increased in the last decade. Our results confirm the importance of sepsis as a significant healthcare issue in Brazil.

**Electronic supplementary material:**

The online version of this article (doi:10.1186/s13054-014-0608-8) contains supplementary material, which is available to authorized users.

## Introduction

Sepsis represents a substantial health care and economic burden worldwide. Reported case fatality rates range from 29 to 60% [1-3]. Treatment costs are estimated at $16.7 to $24.3 billion annually in the US [3,4]. Sepsis incidence has increased in the past decades [1,5,6]. In the US, nearly 3% of patients admitted to hospital have sepsis, and half of these patients are treated in the ICU, accounting for approximately 10% of all ICU admissions [4]. Similar rates have been described in other developed countries [7-9].

In contrast, limited information on sepsis epidemiology, in particular sepsis-related mortality and trends, are available in developing countries. A few recent studies on the topic have been conducted in Brazil [10], China [11], Taiwan [12], Slovak Republic [13], Thailand [14] and Colombia [15]. Nevertheless, reported data are usually not representative, being limited to a single hospital or representing data (for example, from ICU-admitted patients only), which cannot be extrapolated to the population. Up to now, limited population-based estimates of sepsis on a national level for these countries have been conducted and the burden of the disease in those parts of the world remains largely uncharacterized [16]. Likewise, the epidemiology of sepsis-related mortality in Brazil has not been reported from a countrywide perspective.

In this study, we investigated the epidemiology of sepsis deaths in Brazil from 2002 to 2010 using data from the Brazilian National Mortality Information System (*Sistema de Informações de Mortalidade* (SIM)). Our aim was to evaluate trends and provide population-based estimates of sepsis-associated mortality during this period.

## Material and methods

### Study design

We conducted a descriptive study of a retrospective cohort of multiple-cause-of-death data registered in the National Mortality Registry.

### Sources of data

Data routinely reported to the SIM for the years 2002 to 2010, extracted on 28 March 2012, were considered for the analysis. SIM is an electronic, case-based national mortality registry, which derives its information from death certificates. Sepsis-related deaths from 2002 to 2010 were identified based on *International Classification of Disease, 10th Revision* (ICD-10) codes reported for each record of the registry. Information about cause of death available on death certificates includes: part 1: the underlying cause-of-death, the immediate cause-of-death, and the sequence of contributing causes from the underlying to the immediate cause; and part 2: any conditions not directly leading to death but contributing to it. Codes recorded as underlying and/or contributing cause-of-death were considered. In Brazil, death certificate information is collected by trained mortality coders (after appropriate training according to the World Health Organization standards). They work in statistical regional offices of the Ministry of Health and use mortality-coding software specifically designed for this purpose. This practice has remained constant since 1996 (when ICD-10 was introduced in Brazil). Population estimates were obtained from the Brazilian Institute of Geography and Statistics (*Instituto Brasileiro de Geografia e Estatística* (IBGE)) [17].

### Case definitions

Sepsis-associated deaths were defined as those in which the following ICD-10 codes were indicated as any of the causes of death in part 1 of the death certificate: A39, A40 and A41 (including their associated subcodes), A02.1, A22.7, A26.7, A32.7, A 39.2, A39.4, A42.7, B00.7, B37.7 (Additional file 1: Table S1). In addition, in order to identify cases of deaths potentially related to severe sepsis, we selected cases with ICD-10 codes related to any bacterial, protozoan, viral or fungal infections with one or more codes related to organ dysfunction in part 1 of the death certificate (as such, cases of deaths potentially related to severe sepsis include cases of sepsis-associated deaths). Presence of organ dysfunction (cardiovascular, hematologic, hepatic, metabolic, neurologic, respiratory and renal) considered codes listed as the underlying or any other cause of death from part 1 of the death certificate (Additional file 1: Table S2). Presence of underlying comorbidities considered codes listed on parts 1 and 2 of the death certificate (Additional file 1: Table S3). These definitions are slightly modified versions of classifications used by other authors in similar studies [1,4-6,16,18].

When two or more codes were reported in a single cause-of-death variable, separated by a space or a symbol, both of them contributed to the analysis. For this reason, when adding the number of the total reported number of organ failures or comorbidities, more than four may have been reported, despite four being the number of lines available in the death certificate to report immediate and contributing causes-of-death.

### Exclusion criteria

Neonatal and puerperal sepsis-associated deaths (ICD-10 codes P36 and O85, respectively) were excluded from this analysis, as their causes, epidemiological features, prevention and control strategies are different to those related to general sepsis.

### Data analysis

From all deaths recorded in the registry during the study period, all cases of sepsis-associated deaths and deaths potentially related to severe sepsis were identified. The proportion of sepsis deaths for the study period and for every 2-year period was calculated considering the number of sepsis-associated deaths in the numerator and the total number of deaths in Brazil in the denominator.

Annual population-based mortality rates were calculated for the study period, considering the number of sepsis-associated deaths and deaths potentially related to severe sepsis as numerators and the total Brazilian population by year in the denominator. We used the direct standardization method to standardize mortality rates in order to compare them over time. Crude and standardized annual mortality rates were calculated for the period of 2002 to 2010, considering the Brazilian 2010 Census age-stratified population as the standard. We describe the proportion of sepsis-associated deaths by gender, age group, place of occurrence, presence of comorbidities and presence of organ dysfunction. We also tested the association between the above variables and sepsis-associated deaths for the year of 2010.

We used Poisson regression with a logarithmic link function and an offset equal to the log of the population to explore the effects of age group and gender on sepsis-associated deaths mortality rates. Multivariate analysis was used to control for confounders and test for interactions. *P*-values <0.05 were considered statistically significant. However, as the analyses were done with national data and therefore include a large number of events, even small differences can become significant. Thus, when a significant interaction was observed, we decided to report the stratified rates only when the interaction term represented 10% or more of the combined effect of its variables. Analyses were done using Stata 12 (Stata Corporation, College Station, Texas, USA).

Ethical approval for this study was obtained from the Ethical Committee of Hospital Sírio-Libanês, Sao Paulo, Brazil (CAAE: 01063212.1.0000.5461 - 3 January 2012).

## Results

**Figure 1:**
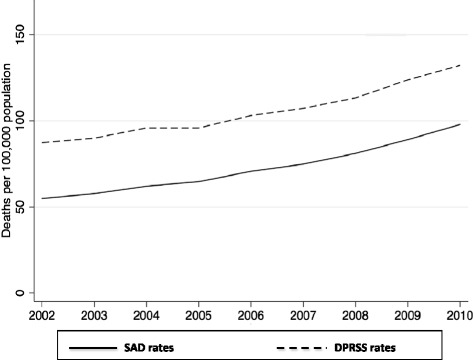
**Rates of sepsis-associated deaths (SAD) and deaths potentially related to severe sepsis (DPRSS).** Brazil, 2002 to 2010.

We identified a total of 9,407,764 deaths in Brazil from 2002 to 2010. The annual number of deaths recorded in SIM increased over time. While in 2002, 982,294 deaths were reported, in 2010 this number increased to 1,133,761 deaths. Similarly, there is an increase in the number of sepsis-associated deaths and deaths potentially related to severe sepsis during the entire period (Figure 1). The proportion of sepsis-associated deaths relative to the total of deaths also increased significantly from 9.77% in 2002 to 16.46% in 2010 (Table 1).

**Table 1:** **Number of total deaths, sepsis-associated deaths, deaths potentially related to severe sepsis, and population during the study period**

**Year**	**Total deaths**	**SAD**	**DPRSS**	**Population**	**% SAD/total deaths**
2002	982,294	95,972	152,482	174,632,932	9.77
2003	1,002,340	102,065	159,181	176,876,251	10.18
2004	1,024,073	111,202	171,751	179,108,134	10.85
2005	1,006,828	119,184	176,643	184,184,074	11.83
2006	1,031,150	131,951	192,475	186,770,613	12.79
2007	1,047,528	141,709	203,138	189,335,191	13.52
2008	1,077,007	153,748	214,964	189,612,814	14.27
2009	1,102,783	170,668	237,032	191,481,045	15.47
2010	1,133,761	186,712	252,263	190,755,799	16.46
Trend *P*-values	NA	0.004	0.004	NA	0.003

Brazil, 2002 to 2010. SAD, sepsis-associated deaths; DPRSS, deaths potentially related to severe sepsis. NA, not applicable.

ICD-10 code A41.9 for Septicemia unspecified was indicated in 98.75% of all sepsis-associated deaths. Chapter XVIII codes R65.1 (Systemic inflammatory response syndrome (SIRS)) and R65.2 (Severe sepsis) were not reported in the SIM database during the study period.

Reported characteristics of sepsis-associated deaths by period are shown in Table 2. The vast majority of sepsis deaths occurred in the hospital. More than two thirds of the patients who died with sepsis had one comorbidity or no comorbidities. Of these, cancer, *diabetes mellitus* and arterial hypertension were the most frequent. Roughly 20% of the sepsis-associated deaths were in patients with had at least one organ failure, the respiratory system being the most frequent dysfunction.

**Table 2:** **Reported characteristics of sepsis-associated deaths**

**Characteristics**	**Values**
Age, years, mean ± SD	64.4 ± 23.4
Male sex, %	51.1
Died in hospital, %	94.8
Comorbidities, %	
Cancer	14.6
*Diabetes mellitus*	13.3
Arterial hypertension	11.0
Stroke	6.3
Renal failure	5.8
Chronic obstructive pulmonary disease	5.7
Congestive heart disease	5.2
Coronary artery disease	3.4
Hepatic condition	2.3
HIV infection	2.5
Alcohol-derived diseases	2.0
Number of comorbidities, %	
0	46.3
1	38.4
2	12.6
3	2.5
4+	0.2
Organ failure, %	
Respiratory	13.5
Renal	4.5
Cardiac	2.4
Hepatic	0.9
Neurologic	0.6
Metabolic	0.5
Hematological	0.3
Number of organ failures, %	
0	79.0
1	19.4
2	1.5
3+	0.1

Brazil, 2002 to 2010.

**Figure 2:**
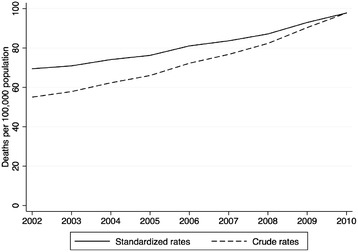
**Crude and age-standardized (using the 2010 Census population as the standard) sepsis-associated mortality rates per 100,000 population.** Brazil, 2002 to 2010.

Crude sepsis-associated mortality rates increased 77.5% during the study period, from 55.1 in 2002 to 97.8/100,000 population in 2010. After age standardization, mortality rates increased 40.7% from 69.5 in 2002 to 97.8 deaths per 100,000 population in 2010. This is equivalent to a linear increase of 3.5 deaths/100,000 population per year (*P* <0.001) (Figure 2).

**Figure 3:**
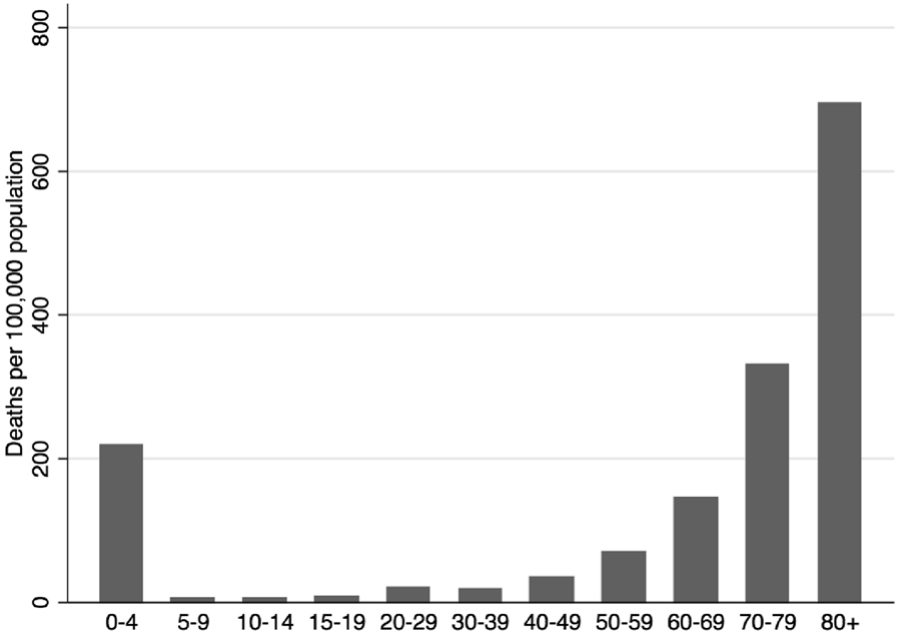
**Crude sepsis-associated mortality rates by age-group.** Brazil, 2002 to 2010.

When analyzing crude sepsis-associated deaths rates by age group, two peaks were demonstrated, the first corresponding to deaths in early childhood, the second in the elderly (Figure 3).

Table 3 shows the crude and adjusted rate ratios of sepsis-associated deaths for age groups and gender, considering all study years. Sepsis-associated mortality increased significantly by age group, being particularly higher in the elderly (60 years and older). Also, sepsis-associated mortality is significantly higher (15%) in men when compared to women.

**Table 3:** **Sepsis-associated mortality rates and rate ratios, by age-group and gender**

**Characteristic**	**Sepsis-associated mortality rates per 100,000 population**	**Crude rate ratios (95% CI)**	**Adjusted rate ratios (95% CI)**
Age groups			
0 to 19 years (ref)	19.3	1.0	1.0
20 to 60 years	34.2	1.77 (1.76, 1.79)	1.78 (1.76, 1.79)
60+ years	297.3	15.6 (15.5, 15.7)	15.7 (15.6, 15.8)
Gender			
Female (ref)	67.8	1.0	1.0
Male	78.2	1.08 (1.07, 1.08)	1.15 (1.15, 1.16)

Brazil, 2002 to 2010. ref, reference.

## Discussion

To our knowledge, this is the first longitudinal, population-based study of deaths associated with sepsis in Brazil. We found that 12.9% of all Brazilian deaths from 2002 to 2010 were related to sepsis, and that both crude and age-standardized sepsis-associated mortality rates were increasing significantly in this period of time. Two peaks of sepsis-related mortality corresponding to early childhood and in the elderly were observed. Moreover, sepsis-associated mortality rates were higher for men.

Sepsis burden is significant both in developed [1-8] and developing countries [10-15]. Our data demonstrate an increase in sepsis-associated deaths (absolute increase of 6.7%) in eight years. Other authors have demonstrated a similar increase in the number of sepsis-associated deaths using hospitalization databases in the US [1,5,6]. While Melamed *et al*. demonstrated a crude sepsis mortality rate of 50.49/100,000 population in 1999 to 2005 [5], Lagu *et al*. reported rates of 87/100,000 in 2007 [3], also using secondary data from hospitalization databases. This represents an increase of 72% in mortality rates, which may be related to increasing trends in sepsis mortality rates in the US. Similarly, we observed an increase of 77.5% in an equivalent period of time in Brazil.

Several authors demonstrated that sepsis-associated mortality rates, the incidence of sepsis and the hospitalizations due to sepsis are increasing [1-3,5,6]. However, age-adjusted case-fatality rates have been decreasing over time in several studies conducted in developed countries, possibly reflecting improvement in access to healthcare and better management of sepsis admitted to hospitals [1,3,5]. Recently, studies reported that better compliance with guidelines for sepsis treatment was associated with improved outcomes [19-21]. This might explain why case-fatality is decreasing. Another issue acknowledged by Lagu *et al*. is the difference in coding of sepsis and organ dysfunction by hospitals when filling hospitalization admission and discharge reports, due to financial issues [3]. As some of these databases are used for reimbursement purposes, this might be linked to financial incentives for coding organ dysfunctions, leading to an increased observed incidence of mild dysfunctions [3]. Our analysis was conducted using data obtained from the national mortality registry, which are generated from death certificates and not used for reimbursement purposes. This data source has fewer fields to input information (in comparison to hospital databases), with no direct relation to clinical data. This might explain why most sepsis-associated deaths had no organ failure reported, which is unlikely, as probably cases of sepsis will have some organ failure at some point over the course of the disease leading to death. This is clearly a restriction of administrative databases and, as such, a limitation of the present study.

Previous studies conducted in Brazil analyzing ICU patients provided relevant information on some epidemiologic aspects of sepsis. The BASES study, which evaluated five Brazilian ICUs in 2001 to 2002, observed 30.5 cases of sepsis/100 ICU admissions. The overall mortality rate for sepsis was 21.8%, and it was 52.2% for patients with septic shock [10]. In another study, 13.4% of ICU admissions were due to severe sepsis, with mortality rates as high as 59.4% [22]. Not only are sepsis ICU admission rates high, but so too are the reported associated costs. Sogayar *et al*. showed a median ICU cost of U$9,632 per patient in a Brazilian study conducted in 21 mixed ICUs in private and public hospitals [23]. To date, trends in the nationwide burden of sepsis over time have not been reported in Brazil. All Brazilian sepsis-related studies have only been done in ICU patients, which might underestimate the overall epidemiologic burden of sepsis in the country. Rezende *et al*. has demonstrated that only one third of patients with severe sepsis admitted to the emergency department of a public tertiary urban hospital in Brazil were referred to an ICU [24]. This suggests that the true sepsis prevalence in hospitals may be much higher than the one observed in ICU only. Resource constraints such as scarcity of ICU beds and high ICU-related costs could be some of the factors that might explain why septic patients are not always admitted to an ICU. The consequence of this fact may be the higher mortality due to sepsis in Brazil, as demonstrated in previous studies [25]. The results obtained in this study, due to its population characteristic, are significant to unequivocally characterize the importance of the disease nationwide.

A recent study evaluated sepsis-associated mortality in England using data from a national mortality database comprising data derived from death certificates [26]. The authors observed that 4.7% of deaths between 2001 and 2010 were associated with sepsis (compared to 12.9% in our study in an equivalent period of time). Similarly to our study, mortality rates were higher in men and increased by age group, with the most significant increase in the elderly. There are relevant advantages of using nationwide routinely collected data such as in our study and the English study for assessing the epidemiology of sepsis. First, long time-series data are readily available for analysis. Second, these data are usually representative of the whole population, and therefore provide a better picture of the nationwide situation. The main disadvantage of using such data sources is that nationwide population-based databases, hospitalization and mortality, are mainly collected for administrative purposes. As such, they tend to have some degree of incompleteness and are more subject to systematic and random errors when compared with data from observational studies. Our data on the low prevalence of organ dysfunction in sepsis-associated deaths from our database is an example of these possible limitations.

In this study, we found an increase in sepsis mortality with age after the first years of life, especially in the elderly subgroup. Others authors have previously demonstrated a similar increase in incidence and mortality of sepsis related to age, reinforcing the generalizability of our findings [4,5,12]. Since there is now an escalation in the proportion of older patients in our country, and the mortality is higher in this subgroup, a further increment in sepsis-associated mortality rates might be expected in the next few years. It should be acknowledged that this change in life expectation might also increase the population at higher risk of death after a septic episode, which could be one of the reasons for the increment in sepsis-associated deaths observed in our study.

Besides the limitations described previously, another possible explanation for the increase in sepsis deaths over time observed in our study is an improvement in notifications of sepsis in death certificates and in data acquisition. Rhee *et al*. recently discussed this possibility, emphasizing the lack of reliable tools to measure sepsis incidence. More important, they suggested that hospitalization rates of sepsis in America are increasing, but infection rates are stable or decreasing. This may be due to differences in coding practice associated with changes in reimbursement values instead of a true increase in sepsis rates [27]. However, in Brazil, there are no major modifications in reimbursement values for coding associated with organ dysfunction. In addition, we searched for pneumonia-associated deaths in the same period in our country and we observed an increase at a similar rate to sepsis-associated deaths (data not shown). This is in clear contrast to Rhee’s data, and reinforces the burden of infection-associated deaths in Brazil. In fact, it is possible that our results still underestimate the real burden of sepsis, as death certificate analysis might underestimate infections as a cause of death [28]. Finally, another limitation of our study is the data abstraction using ICD-10 coding from death certificates. Other approaches (such as Angus Implementation) are available, but inaccuracies are still present [29,30]. On the other hand, previous studies using death certificates have been published with results similar to ours [6,26], strengthening our findings.

## Conclusions

In conclusion, sepsis-associated mortality in Brazil is relevant due to its increasing number of deaths in recent years, a trend not related solely to the aging population. Our data confirm the importance of sepsis as a significant healthcare issue in Brazil.

## Key messages

- This is the first longitudinal, population-based study of sepsis-associated deaths in Brazil
- From 2002 to 2010, 12.9% of all Brazilian deaths were related to sepsis and both crude and age-standardized sepsis associated mortality rates are increasing
- In Brazil, sepsis-related deaths are higher in early childhood and the elderly, and males have higher rates of death than females.

## Additional file

Additional file 1: Table S1. International Classification of Diseases 10th Revision (ICD-10) codes used for definition of sepsis-associated deaths. **Table S2.** International Classification of Diseases 10th Revision (ICD-10) codes used for definition of organ dysfunction. **Table S3.** International Classification of Diseases 10th Revision (ICD-10) codes used for definition of comorbidity. **Table S4.** International Classification of Diseases 10th Revision (ICD-10) codes used for definition of infection (bacterial, protozoan, viral or fungal).

## Abbreviations

DPRSS: deaths potentially related to severe sepsis
ICD-10: International Classification of Diseases, 10th Revision
SAD: sepsis-associated deaths
SIM: *Sistema de Informações de Mortalidade* (Brazilian National Mortality Information System)
