# Supplementary material for: Sepsis-related deaths in Brazil: an analysis of the national mortality registry from 2002 to 2010
Source: Crit Care. 2014 Nov 5;18(6):608. doi: 10.1186/s13054-014-0608-8 (PMC4240892; doi:10.1186/s13054-014-0608-8)
Supplement: Additional file 1: Table S1. — International Classification of Diseases 10th Revision (ICD-10) codes used for definition of sepsis-associated deaths. Table S2. International Classification of Diseases 10th Revision (ICD-10) codes used for definition of organ dysfunction. Table S3. International Classification of Diseases 10th Revision (ICD-10) codes used for definition of comorbidity. Table S4. International Classification of Diseases 10th Revision (ICD-10) codes used for definition of infection (bacterial, protozoan, viral or fungal). [file 13054_2014_608_MOESM1_ESM.docx]

**Electronic Supplement**

# Sepsis-related deaths in Brazil: An analysis of the National mortality registry from 2002 to 2010

Leandro U. Taniguchi^1,2^, Ana Luiza Bierrenbach^1,3^, Cristiana M. Toscano^1,4^, Guilherme P. P. Schettino^1^, Luciano C. P. Azevedo^1,2^

^1^ Research and Education Institute (IEP), Hospital Sirio-Libanes, Rua Cel. Nicolau dos Santos 69, São Paulo, Brazil.

^2^ Emergency Medicine Discipline, Hospital das Clínicas da Faculdade de Medicina da Universidade de São Paulo, Av Enéas de Carvalho Aguiar 255 Sala 5023, São Paulo, Brazil.

^3^ Sanas Epidemiology and Research, Avenida Paulista 2073, Edifício Horsa 1, salas 703/704, São Paulo, Brazil

^4^ Department of Collective Health, Federal University of Goias, Rua 235 s/n, Goias, Brazil.

Author for correspondence:

Luciano C. P. Azevedo

Research and Education Institute, Hospital Sirio-Libanes, São Paulo, Brazil.

Rua Cel. Nicolau dos Santos, 69. São Paulo – Brazil. ZIP: 01308-060

Phone: +551131553706; email: luciano.azevedo@hsl.org.br

In the tables below, where 3- or 4-digit codes are listed, all associated subcodes were included.

Table S1. International Classification of Diseases 10th Revision (ICD-10) codes used for sepsis associated deaths definition

| **ICD-10 code** | **Description** |
| --- | --- |
| A39 (and associated subcodes) | Meningococcal meningitis |
| A40 (and associated subcodes) | Streptococcal sepsis |
| A41 (and associated subcodes) | Other sepsis |
| A02.1 | Salmonella sepsis |
| A22.7 | Anthrax sepsis |
| A26.7 | Erysipelothrix sepsis |
| A32.7 | Listeria sepsis |
| A39.2 | Acute meningococcemia |
| A39.4 | Meningococcemia, unspecified |
| A42.7 | Actinomycotic sepsis |
| B00.7 | Disseminated herpesvirus disease |
| B37.7 | Candida sepsis |

Table S2. International Classification of Diseases 10th Revision (ICD-10) codes used for organ dysfunction definition

| **ICD-10 code** | **Description** | **Organ failure** |
| --- | --- | --- |
| R570 | Cardiogenic shock | cardiovascular |
| R578 | Other shock | cardiovascular |
| R579 | Shock unspecified | cardiovascular |
| D65 | Disseminated intravascular coagulation | hematologic |
| D689 | Coagulation defect unspecified | hematologic |
| D695 | Secondary thrombocytopenia | hematologic |
| D696 | Thrombocytopenia unspecified | hematologic |
| K720 | Acute and subacute hepatic failure | hepatic |
| K729 | Hepatic failure unspecified | hepatic |
| K763 | Infarction of liver | hepatic |
| E86 | Volume depletion | metabolic |
| E872 | Acidosis | metabolic |
| E874 | Mixed disorder of acidbase balance | metabolic |
| F058 | Other delirium | neurologic |
| F059 | Delirium unspecified | neurologic |
| G931 | Anoxic brain damage not elsewhere classified | neurologic |
| G934 | Encephalopathy unspecified | neurologic |
| R400 | Somnolence | neurologic |
| R401 | Stupor | neurologic |
| R402 | Coma unspecified | neurologic |
| R410 | Disorientation unspecified | neurologic |
| N170 | Acute renal failure with tubular necrosis | renal |
| N171 | Acute renal failure with acute cortical necrosis | renal |
| N172 | Acute renal failure with medullary necrosis | renal |
| N178 | Other acute renal failure | renal |
| N179 | Acute renal failure unspecified | renal |
| N19 | Unspecified renal failure | renal |
| J80 | Adult respiratory distress syndrome | respiratory |
| J960 | Acute respiratory failure | respiratory |
| J969 | Respiratory failure unspecified | respiratory |
| U049 | Severe acute respiratory syndrome [SARS] unspecified | respiratory |

Table S3. International Classification of Diseases 10th Revision (ICD-10) codes used for comorbidity definition

| **ICD-10 code** | **Description** | **Comorbidity** |
| --- | --- | --- |
| F102 | Mental and behavioural disorders due to use of alcohol dependence syndrome | alcohol |
| G312 | Degeneration of nervous system due to alcohol | alcohol |
| G621 | Alcoholic polyneuropathy | alcohol |
| G721 | Alcoholic myopathy | alcohol |
| I426 | Alcoholic cardiomyopathy | alcohol |
| K292 | Alcoholic gastritis | alcohol |
| K860 | Alcoholinduced chronic pancreatitis | alcohol |
| C000 | External upper lip | cancer |
| C001 | External lower lip | cancer |
| C002 | External lip unspecified | cancer |
| C003 | Upper lip inner aspect | cancer |
| C004 | Lower lip inner aspect | cancer |
| C005 | Lip unspecified inner aspect | cancer |
| C006 | Commissure of lip | cancer |
| C008 | Overlapping lesion of lip | cancer |
| C009 | Lip unspecified | cancer |
| C01 | Malignant neoplasm of base of tongue | cancer |
| C020 | Dorsal surface of tongue | cancer |
| C021 | Border of tongue | cancer |
| C022 | Ventral surface of tongue | cancer |
| C023 | Anterior twothirds of tongue part unspecified | cancer |
| C024 | Lingual tonsil | cancer |
| C028 | Overlapping lesion of tongue | cancer |
| C029 | Tongue unspecified | cancer |
| C030 | Upper gum | cancer |
| C031 | Lower gum | cancer |
| C039 | Gum unspecified | cancer |
| C040 | Anterior floor of mouth | cancer |
| C041 | Lateral floor of mouth | cancer |
| C048 | Overlapping lesion of floor of mouth | cancer |
| C049 | Floor of mouth unspecified | cancer |
| C050 | Hard palate | cancer |
| C051 | Soft palate | cancer |
| C052 | Uvula | cancer |
| C058 | Overlapping lesion of palate | cancer |
| C059 | Palate unspecified | cancer |
| C060 | Cheek mucosa | cancer |
| C061 | Vestibule of mouth | cancer |
| C062 | Retromolar area | cancer |
| C068 | Overlapping lesion of other and unspecified parts of mouth | cancer |
| C069 | Mouth unspecified | cancer |
| C07 | Malignant neoplasm of parotid gland | cancer |
| C080 | Submandibular gland | cancer |
| C081 | Sublingual gland | cancer |
| C088 | Overlapping lesion of major salivary glands | cancer |
| C089 | Major salivary gland unspecified | cancer |
| C090 | Tonsillar fossa | cancer |
| C091 | Tonsillar pillar (anterior)(posterior) | cancer |
| C098 | Overlapping lesion of tonsil | cancer |
| C099 | Tonsil unspecified | cancer |
| C100 | Vallecula | cancer |
| C101 | Anterior surface of epiglottis | cancer |
| C102 | Lateral wall of oropharynx | cancer |
| C103 | Posterior wall of oropharynx | cancer |
| C104 | Branchial cleft | cancer |
| C108 | Overlapping lesion of oropharynx | cancer |
| C109 | Oropharynx unspecified | cancer |
| C110 | Superior wall of nasopharynx | cancer |
| C111 | Posterior wall of nasopharynx | cancer |
| C112 | Lateral wall of nasopharynx | cancer |
| C113 | Anterior wall of nasopharynx | cancer |
| C118 | Overlapping lesion of nasopharynx | cancer |
| C119 | Nasopharynx unspecified | cancer |
| C12 | Malignant neoplasm of pyriform sinus | cancer |
| C130 | Postcricoid region | cancer |
| C131 | Aryepiglottic fold hypopharyngeal aspect | cancer |
| C132 | Posterior wall of hypopharynx | cancer |
| C138 | Overlapping lesion of hypopharynx | cancer |
| C139 | Hypopharynx unspecified | cancer |
| C140 | Pharynx unspecified | cancer |
| C142 | Waldeyer's ring | cancer |
| C148 | Overlapping lesion of lip oral cavity and pharynx | cancer |
| C150 | Cervical part of oesophagus | cancer |
| C151 | Thoracic part of oesophagus | cancer |
| C152 | Abdominal part of oesophagus | cancer |
| C153 | Upper third of oesophagus | cancer |
| C154 | Middle third of oesophagus | cancer |
| C155 | Lower third of oesophagus | cancer |
| C158 | Overlapping lesion of oesophagus | cancer |
| C159 | Oesophagus unspecified | cancer |
| C160 | Cardia | cancer |
| C161 | Fundus of stomach | cancer |
| C162 | Body of stomach | cancer |
| C163 | Pyloric antrum | cancer |
| C164 | Pylorus | cancer |
| C165 | Lesser curvature of stomach unspecified | cancer |
| C166 | Greater curvature of stomach unspecified | cancer |
| C168 | Overlapping lesion of stomach | cancer |
| C169 | Stomach unspecified | cancer |
| C170 | Duodenum | cancer |
| C171 | Jejunum | cancer |
| C172 | Ileum | cancer |
| C173 | Meckel's diverticulum | cancer |
| C178 | Overlapping lesion of small intestine | cancer |
| C179 | Small intestine unspecified | cancer |
| C180 | Caecum | cancer |
| C181 | Appendix | cancer |
| C182 | Ascending colon | cancer |
| C183 | Hepatic flexure | cancer |
| C184 | Transverse colon | cancer |
| C185 | Splenic flexure | cancer |
| C186 | Descending colon | cancer |
| C187 | Sigmoid colon | cancer |
| C188 | Overlapping lesion of colon | cancer |
| C189 | Colon unspecified | cancer |
| C19 | Malignant neoplasm of rectosigmoid junction | cancer |
| C20 | Malignant neoplasm of rectum | cancer |
| C210 | Anus unspecified | cancer |
| C211 | Anal canal | cancer |
| C212 | Cloacogenic zone | cancer |
| C218 | Overlapping lesion of rectum anus and anal canal | cancer |
| C220 | Liver cell carcinoma | cancer |
| C221 | Intrahepatic bile duct carcinoma | cancer |
| C222 | Hepatoblastoma | cancer |
| C223 | Angiosarcoma of liver | cancer |
| C224 | Other sarcomas of liver | cancer |
| C227 | Other specified carcinomas of liver | cancer |
| C229 | Liver unspecified | cancer |
| C23 | Malignant neoplasm of gallbladder | cancer |
| C240 | Extrahepatic bile duct | cancer |
| C241 | Ampulla of Vater | cancer |
| C248 | Overlapping lesion of biliary tract | cancer |
| C249 | Biliary tract unspecified | cancer |
| C250 | Head of pancreas | cancer |
| C251 | Body of pancreas | cancer |
| C252 | Tail of pancreas | cancer |
| C253 | Pancreatic duct | cancer |
| C254 | Endocrine pancreas | cancer |
| C257 | Other parts of pancreas | cancer |
| C258 | Overlapping lesion of pancreas | cancer |
| C259 | Pancreas unspecified | cancer |
| C260 | Intestinal tract part unspecified | cancer |
| C261 | Spleen | cancer |
| C268 | Overlapping lesion of digestive system | cancer |
| C269 | Illdefined sites within the digestive system | cancer |
| C300 | Nasal cavity | cancer |
| C301 | Middle ear | cancer |
| C310 | Maxillary sinus | cancer |
| C311 | Ethmoidal sinus | cancer |
| C312 | Frontal sinus | cancer |
| C313 | Sphenoidal sinus | cancer |
| C318 | Overlapping lesion of accessory sinuses | cancer |
| C319 | Accessory sinus unspecified | cancer |
| C320 | Glottis | cancer |
| C321 | Supraglottis | cancer |
| C322 | Subglottis | cancer |
| C323 | Laryngeal cartilage | cancer |
| C328 | Overlapping lesion of larynx | cancer |
| C329 | Larynx unspecified | cancer |
| C33 | Malignant neoplasm of trachea | cancer |
| C340 | Main bronchus | cancer |
| C341 | Upper lobe bronchus or lung | cancer |
| C342 | Middle lobe bronchus or lung | cancer |
| C343 | Lower lobe bronchus or lung | cancer |
| C348 | Overlapping lesion of bronchus and lung | cancer |
| C349 | Bronchus or lung unspecified | cancer |
| C37 | Malignant neoplasm of thymus | cancer |
| C380 | Heart | cancer |
| C381 | Anterior mediastinum | cancer |
| C382 | Posterior mediastinum | cancer |
| C383 | Mediastinum part unspecified | cancer |
| C384 | Pleura | cancer |
| C388 | Overlapping lesion of heart mediastinum and pleura | cancer |
| C390 | Upper respiratory tract part unspecified | cancer |
| C398 | Overlapping lesion of respiratory and intrathoracic organs | cancer |
| C399 | Illdefined sites within the respiratory system | cancer |
| C400 | Scapula and long bones of upper limb | cancer |
| C401 | Short bones of upper limb | cancer |
| C402 | Long bones of lower limb | cancer |
| C403 | Short bones of lower limb | cancer |
| C408 | Overlapping lesion of bone and articular cartilage of limbs | cancer |
| C409 | Bone and articular cartilage of limb unspecified | cancer |
| C410 | Bones of skull and face | cancer |
| C411 | Mandible | cancer |
| C412 | Vertebral column | cancer |
| C413 | Ribs sternum and clavicle | cancer |
| C414 | Pelvic bones sacrum and coccyx | cancer |
| C418 | Overlapping lesion of bone and articular cartilage | cancer |
| C419 | Bone and articular cartilage unspecified | cancer |
| C430 | Malignant melanoma of lip | cancer |
| C431 | Malignant melanoma of eyelid including canthus | cancer |
| C432 | Malignant melanoma of ear and external auricular canal | cancer |
| C433 | Malignant melanoma of other and unspecified parts of face | cancer |
| C434 | Malignant melanoma of scalp and neck | cancer |
| C435 | Malignant melanoma of trunk | cancer |
| C436 | Malignant melanoma of upper limb including shoulder | cancer |
| C437 | Malignant melanoma of lower limb including hip | cancer |
| C438 | Overlapping malignant melanoma of skin | cancer |
| C439 | Malignant melanoma of skin unspecified | cancer |
| C440 | Skin of lip | cancer |
| C441 | Skin of eyelid including canthus | cancer |
| C442 | Skin of ear and external auricular canal | cancer |
| C443 | Skin of other and unspecified parts of face | cancer |
| C444 | Skin of scalp and neck | cancer |
| C445 | Skin of trunk | cancer |
| C446 | Skin of upper limb including shoulder | cancer |
| C447 | Skin of lower limb including hip | cancer |
| C448 | Overlapping lesion of skin | cancer |
| C449 | Malignant neoplasm of skin unspecified | cancer |
| C450 | Mesothelioma of pleura | cancer |
| C451 | Mesothelioma of peritoneum | cancer |
| C452 | Mesothelioma of pericardium | cancer |
| C457 | Mesothelioma of other sites | cancer |
| C459 | Mesothelioma unspecified | cancer |
| C460 | Kaposi's sarcoma of skin | cancer |
| C461 | Kaposi's sarcoma of soft tissue | cancer |
| C462 | Kaposi's sarcoma of palate | cancer |
| C463 | Kaposi's sarcoma of lymph nodes | cancer |
| C467 | Kaposi's sarcoma of other sites | cancer |
| C468 | Kaposi's sarcoma of multiple organs | cancer |
| C469 | Kaposi's sarcoma unspecified | cancer |
| C470 | Peripheral nerves of head face and neck | cancer |
| C471 | Peripheral nerves of upper limb including shoulder | cancer |
| C472 | Peripheral nerves of lower limb including hip | cancer |
| C473 | Peripheral nerves of thorax | cancer |
| C474 | Peripheral nerves of abdomen | cancer |
| C475 | Peripheral nerves of pelvis | cancer |
| C476 | Peripheral nerves of trunk unspecified | cancer |
| C478 | Overlapping lesion of peripheral nerves and autonomic nervous system | cancer |
| C479 | Peripheral nerves and autonomic nervous system unspecified | cancer |
| C480 | Retroperitoneum | cancer |
| C481 | Specified parts of peritoneum | cancer |
| C482 | Peritoneum unspecified | cancer |
| C488 | Overlapping lesion of retroperitoneum and peritoneum | cancer |
| C490 | Connective and soft tissue of head face and neck | cancer |
| C491 | Connective and soft tissue of upper limb including shoulder | cancer |
| C492 | Connective and soft tissue of lower limb including hip | cancer |
| C493 | Connective and soft tissue of thorax | cancer |
| C494 | Connective and soft tissue of abdomen | cancer |
| C495 | Connective and soft tissue of pelvis | cancer |
| C496 | Connective and soft tissue of trunk unspecified | cancer |
| C498 | Overlapping lesion of connective and soft tissue | cancer |
| C499 | Connective and soft tissue unspecified | cancer |
| C500 | Nipple and areola | cancer |
| C501 | Central portion of breast | cancer |
| C502 | Upperinner quadrant of breast | cancer |
| C503 | Lowerinner quadrant of breast | cancer |
| C504 | Upperouter quadrant of breast | cancer |
| C505 | Lowerouter quadrant of breast | cancer |
| C506 | Axillary tail of breast | cancer |
| C508 | Overlapping lesion of breast | cancer |
| C509 | Breast unspecified | cancer |
| C510 | Labium majus | cancer |
| C511 | Labium minus | cancer |
| C512 | Clitoris | cancer |
| C518 | Overlapping lesion of vulva | cancer |
| C519 | Vulva unspecified | cancer |
| C52 | Malignant neoplasm of vagina | cancer |
| C530 | Endocervix | cancer |
| C531 | Exocervix | cancer |
| C538 | Overlapping lesion of cervix uteri | cancer |
| C539 | Cervix uteri unspecified | cancer |
| C540 | Isthmus uteri | cancer |
| C541 | Endometrium | cancer |
| C542 | Myometrium | cancer |
| C543 | Fundus uteri | cancer |
| C548 | Overlapping lesion of corpus uteri | cancer |
| C549 | Corpus uteri unspecified | cancer |
| C55 | Malignant neoplasm of uterus part unspecified | cancer |
| C56 | Malignant neoplasm of ovary | cancer |
| C570 | Fallopian tube | cancer |
| C571 | Broad ligament | cancer |
| C572 | Round ligament | cancer |
| C573 | Parametrium | cancer |
| C574 | Uterine adnexa unspecified | cancer |
| C577 | Other specified female genital organs | cancer |
| C578 | Overlapping lesion of female genital organs | cancer |
| C579 | Female genital organ unspecified | cancer |
| C58 | Malignant neoplasm of placenta | cancer |
| C600 | Prepuce | cancer |
| C601 | Glans penis | cancer |
| C602 | Body of penis | cancer |
| C608 | Overlapping lesion of penis | cancer |
| C609 | Penis unspecified | cancer |
| C61 | Malignant neoplasm of prostate | cancer |
| C620 | Undescended testis | cancer |
| C621 | Descended testis | cancer |
| C629 | Testis unspecified | cancer |
| C630 | Epididymis | cancer |
| C631 | Spermatic cord | cancer |
| C632 | Scrotum | cancer |
| C637 | Other specified male genital organs | cancer |
| C638 | Overlapping lesion of male genital organs | cancer |
| C639 | Male genital organ unspecified | cancer |
| C64 | Malignant neoplasm of kidney except renal pelvis | cancer |
| C65 | Malignant neoplasm of renal pelvis | cancer |
| C66 | Malignant neoplasm of ureter | cancer |
| C670 | Trigone of bladder | cancer |
| C671 | Dome of bladder | cancer |
| C672 | Lateral wall of bladder | cancer |
| C673 | Anterior wall of bladder | cancer |
| C674 | Posterior wall of bladder | cancer |
| C675 | Bladder neck | cancer |
| C676 | Ureteric orifice | cancer |
| C677 | Urachus | cancer |
| C678 | Overlapping lesion of bladder | cancer |
| C679 | Bladder unspecified | cancer |
| C680 | Urethra | cancer |
| C681 | Paraurethral gland | cancer |
| C688 | Overlapping lesion of urinary organs | cancer |
| C689 | Urinary organ unspecified | cancer |
| C690 | Conjunctiva | cancer |
| C691 | Cornea | cancer |
| C692 | Retina | cancer |
| C693 | Choroid | cancer |
| C694 | Ciliary body | cancer |
| C695 | Lacrimal gland and duct | cancer |
| C696 | Orbit | cancer |
| C698 | Overlapping lesion of eye and adnexa | cancer |
| C699 | Eye unspecified | cancer |
| C700 | Cerebral meninges | cancer |
| C701 | Spinal meninges | cancer |
| C709 | Meninges unspecified | cancer |
| C710 | Cerebrum except lobes and ventricles | cancer |
| C711 | Frontal lobe | cancer |
| C712 | Temporal lobe | cancer |
| C713 | Parietal lobe | cancer |
| C714 | Occipital lobe | cancer |
| C715 | Cerebral ventricle | cancer |
| C716 | Cerebellum | cancer |
| C717 | Brain stem | cancer |
| C718 | Overlapping lesion of brain | cancer |
| C719 | Brain unspecified | cancer |
| C720 | Spinal cord | cancer |
| C721 | Cauda equina | cancer |
| C722 | Olfactory nerve | cancer |
| C723 | Optic nerve | cancer |
| C724 | Acoustic nerve | cancer |
| C725 | Other and unspecified cranial nerves | cancer |
| C728 | Overlapping lesion of brain and other parts of central nervous system | cancer |
| C729 | Central nervous system unspecified | cancer |
| C73 | Malignant neoplasm of thyroid gland | cancer |
| C740 | Cortex of adrenal gland | cancer |
| C741 | Medulla of adrenal gland | cancer |
| C749 | Adrenal gland unspecified | cancer |
| C750 | Parathyroid gland | cancer |
| C751 | Pituitary gland | cancer |
| C752 | Craniopharyngeal duct | cancer |
| C753 | Pineal gland | cancer |
| C754 | Carotid body | cancer |
| C755 | Aortic body and other paraganglia | cancer |
| C758 | Pluriglandular involvement unspecified | cancer |
| C759 | Endocrine gland unspecified | cancer |
| C760 | Head face and neck | cancer |
| C761 | Thorax | cancer |
| C762 | Abdomen | cancer |
| C763 | Pelvis | cancer |
| C764 | Upper limb | cancer |
| C765 | Lower limb | cancer |
| C767 | Other illdefined sites | cancer |
| C768 | Overlapping lesion of other and illdefined sites | cancer |
| C770 | Lymph nodes of head face and neck | cancer |
| C771 | Intrathoracic lymph nodes | cancer |
| C772 | Intraabdominal lymph nodes | cancer |
| C773 | Axillary and upper limb lymph nodes | cancer |
| C774 | Inguinal and lower limb lymph nodes | cancer |
| C775 | Intrapelvic lymph nodes | cancer |
| C778 | Lymph nodes of multiple regions | cancer |
| C779 | Lymph node unspecified | cancer |
| C780 | Secondary malignant neoplasm of lung | cancer |
| C781 | Secondary malignant neoplasm of mediastinum | cancer |
| C782 | Secondary malignant neoplasm of pleura | cancer |
| C783 | Secondary malignant neoplasm of other and unspecified respiratory organs | cancer |
| C784 | Secondary malignant neoplasm of small intestine | cancer |
| C785 | Secondary malignant neoplasm of large intestine and rectum | cancer |
| C786 | Secondary malignant neoplasm of retroperitoneum and peritoneum | cancer |
| C787 | Secondary malignant neoplasm of liver | cancer |
| C788 | Secondary malignant neoplasm of other and unspecified digestive organs | cancer |
| C790 | Secondary malignant neoplasm of kidney and renal pelvis | cancer |
| C791 | Secondary malignant neoplasm of bladder and other and unspecified urinary organs | cancer |
| C792 | Secondary malignant neoplasm of skin | cancer |
| C793 | Secondary malignant neoplasm of brain and cerebral meninges | cancer |
| C794 | Secondary malignant neoplasm of other and unspecified parts of nervous system | cancer |
| C795 | Secondary malignant neoplasm of bone and bone marrow | cancer |
| C796 | Secondary malignant neoplasm of ovary | cancer |
| C797 | Secondary malignant neoplasm of adrenal gland | cancer |
| C798 | Secondary malignant neoplasm of other specified sites | cancer |
| C80 | Malignant neoplasm without specification of site | cancer |
| C810 | Lymphocytic predominance | cancer |
| C811 | Nodular sclerosis | cancer |
| C812 | Mixed cellularity | cancer |
| C813 | Lymphocytic depletion | cancer |
| C817 | Other Hodgkin's disease | cancer |
| C819 | Hodgkin's disease unspecified | cancer |
| C820 | Small cleaved cell follicular | cancer |
| C821 | Mixed small cleaved and large cell follicular | cancer |
| C822 | Large cell follicular | cancer |
| C827 | Other types of follicular nonHodgkin's lymphoma | cancer |
| C829 | Follicular nonHodgkin's lymphoma unspecified | cancer |
| C830 | Small cell (diffuse) | cancer |
| C831 | Small cleaved cell (diffuse) | cancer |
| C832 | Mixed small and large cell (diffuse) | cancer |
| C833 | Large cell (diffuse) | cancer |
| C834 | Immunoblastic (diffuse) | cancer |
| C835 | Lymphoblastic (diffuse) | cancer |
| C836 | Undifferentiated (diffuse) | cancer |
| C837 | Burkitt's tumour | cancer |
| C838 | Other types of diffuse nonHodgkin's lymphoma | cancer |
| C839 | Diffuse nonHodgkin's lymphoma unspecified | cancer |
| C840 | Mycosis fungoides | cancer |
| C841 | Sézary's disease | cancer |
| C842 | Tzone lymphoma | cancer |
| C843 | Lymphoepithelioid lymphoma | cancer |
| C844 | Peripheral Tcell lymphoma | cancer |
| C845 | Other and unspecified Tcell lymphomas | cancer |
| C850 | Lymphosarcoma | cancer |
| C851 | Bcell lymphoma unspecified | cancer |
| C857 | Other specified types of nonHodgkin's lymphoma | cancer |
| C859 | NonHodgkin's lymphoma unspecified type | cancer |
| C880 | Waldenström's macroglobulinaemia | cancer |
| C881 | Alpha heavy chain disease | cancer |
| C882 | Gamma heavy chain disease | cancer |
| C883 | Immunoproliferative small intestinal disease | cancer |
| C887 | Other malignant immunoproliferative diseases | cancer |
| C889 | Malignant immunoproliferative disease unspecified | cancer |
| C900 | Multiple myeloma | cancer |
| C901 | Plasma cell leukaemia | cancer |
| C902 | Plasmacytoma extramedullary | cancer |
| C910 | Acute lymphoblastic leukaemia | cancer |
| C911 | Chronic lymphocytic leukaemia | cancer |
| C912 | Subacute lymphocytic leukaemia | cancer |
| C913 | Prolymphocytic leukaemia | cancer |
| C914 | Hairycell leukaemia | cancer |
| C915 | Adult Tcell leukaemia | cancer |
| C917 | Other lymphoid leukaemia | cancer |
| C919 | Lymphoid leukaemia unspecified | cancer |
| C920 | Acute myeloid leukaemia | cancer |
| C921 | Chronic myeloid leukaemia | cancer |
| C922 | Subacute myeloid leukaemia | cancer |
| C923 | Myeloid sarcoma | cancer |
| C924 | Acute promyelocytic leukaemia | cancer |
| C925 | Acute myelomonocytic leukaemia | cancer |
| C927 | Other myeloid leukaemia | cancer |
| C929 | Myeloid leukaemia unspecified | cancer |
| C930 | Acute monocytic leukaemia | cancer |
| C931 | Chronic monocytic leukaemia | cancer |
| C932 | Subacute monocytic leukaemia | cancer |
| C937 | Other monocytic leukaemia | cancer |
| C939 | Monocytic leukaemia unspecified | cancer |
| C940 | Acute erythraemia and erythroleukaemia | cancer |
| C941 | Chronic erythraemia | cancer |
| C942 | Acute megakaryoblastic leukaemia | cancer |
| C943 | Mast cell leukaemia | cancer |
| C944 | Acute panmyelosis | cancer |
| C945 | Acute myelofibrosis | cancer |
| C947 | Other specified leukaemias | cancer |
| C950 | Acute leukaemia of unspecified cell type | cancer |
| C951 | Chronic leukaemia of unspecified cell type | cancer |
| C952 | Subacute leukaemia of unspecified cell type | cancer |
| C957 | Other leukaemia of unspecified cell type | cancer |
| C959 | Leukaemia unspecified | cancer |
| C960 | LettererSiwe disease | cancer |
| C961 | Malignant histiocytosis | cancer |
| C962 | Malignant mast cell tumour | cancer |
| C963 | True histiocytic lymphoma | cancer |
| C967 | Other specified malignant neoplasms of lymphoid haematopoietic and related tissue | cancer |
| C969 | Malignant neoplasm of lymphoid haematopoietic and related tissue unspecified | cancer |
| C97 | Malignant neoplasms of independent (primary) multiple sites | cancer |
| I500 | Congestive heart failure | cardiac |
| I501 | Left ventricular failure | cardiac |
| I509 | Heart failure unspecified | cardiac |
| J40 | Bronchitis not specified as acute or chronic | COPD |
| J410 | Simple chronic bronchitis | COPD |
| J411 | Mucopurulent chronic bronchitis | COPD |
| J418 | Mixed simple and mucopurulent chronic bronchitis | COPD |
| J42 | Unspecified chronic bronchitis | COPD |
| J430 | MacLeod's syndrome | COPD |
| J431 | Panlobular emphysema | COPD |
| J432 | Centrilobular emphysema | COPD |
| J438 | Other emphysema | COPD |
| J439 | Emphysema unspecified | COPD |
| J440 | Chronic obstructive pulmonary disease with acute lower respiratory infection | COPD |
| J441 | Chronic obstructive pulmonary disease with acute exacerbation unspecified | COPD |
| J448 | Other specified chronic obstructive pulmonary disease | COPD |
| J449 | Chronic obstructive pulmonary disease unspecified | COPD |
| J961 | Chronic respiratory failure | COPD |
| I200 | Unstable angina | coronary |
| I201 | Angina pectoris with documented spasm | coronary |
| I208 | Other forms of angina pectoris | coronary |
| I209 | Angina pectoris unspecified | coronary |
| I210 | Acute transmural myocardial infarction of anterior wall | coronary |
| I211 | Acute transmural myocardial infarction of inferior wall | coronary |
| I212 | Acute transmural myocardial infarction of other sites | coronary |
| I213 | Acute transmural myocardial infarction of unspecified site | coronary |
| I214 | Acute subendocardial myocardial infarction | coronary |
| I219 | Acute myocardial infarction unspecified | coronary |
| I220 | Subsequent myocardial infarction of anterior wall | coronary |
| I221 | Subsequent myocardial infarction of inferior wall | coronary |
| I228 | Subsequent myocardial infarction of other sites | coronary |
| I229 | Subsequent myocardial infarction of unspecified site | coronary |
| I241 | Dressler's syndrome | coronary |
| I248 | Other forms of acute ischaemic heart disease | coronary |
| I249 | Acute ischaemic heart disease unspecified | coronary |
| I250 | Atherosclerotic cardiovascular disease so described | coronary |
| I251 | Atherosclerotic heart disease | coronary |
| I253 | Aneurysm of heart | coronary |
| I254 | Coronary artery aneurysm | coronary |
| I255 | Ischaemic cardiomyopathy | coronary |
| I256 | Silent myocardial ischaemia | coronary |
| I258 | Other forms of chronic ischaemic heart disease | coronary |
| I259 | Chronic ischaemic heart disease unspecified | coronary |
| E100 | Insulindependent diabetes mellitus with coma | diabetes mellitus |
| E101 | Insulindependent diabetes mellitus with ketoacidosis | diabetes mellitus |
| E102 | Insulindependent diabetes mellitus with renal complications | diabetes mellitus |
| E103 | Insulindependent diabetes mellitus with ophthalmic complications | diabetes mellitus |
| E104 | Insulindependent diabetes mellitus with neurological complications | diabetes mellitus |
| E105 | Insulindependent diabetes mellitus with peripheral circulatory complications | diabetes mellitus |
| E106 | Insulindependent diabetes mellitus with other specified complications | diabetes mellitus |
| E107 | Insulindependent diabetes mellitus with multiple complications | diabetes mellitus |
| E108 | Insulindependent diabetes mellitus with unspecified complications | diabetes mellitus |
| E109 | Insulindependent diabetes mellitus without complications | diabetes mellitus |
| E110 | Noninsulindependent diabetes mellitus with coma | diabetes mellitus |
| E111 | Noninsulindependent diabetes mellitus with ketoacidosis | diabetes mellitus |
| E112 | Noninsulindependent diabetes mellitus with renal complications | diabetes mellitus |
| E113 | Noninsulindependent diabetes mellitus with ophthalmic complications | diabetes mellitus |
| E114 | Noninsulindependent diabetes mellitus with neurological complications | diabetes mellitus |
| E115 | Noninsulindependent diabetes mellitus with peripheral circulatory complications | diabetes mellitus |
| E116 | Noninsulindependent diabetes mellitus with other specified complications | diabetes mellitus |
| E117 | Noninsulindependent diabetes mellitus with multiple complications | diabetes mellitus |
| E118 | Noninsulindependent diabetes mellitus with unspecified complications | diabetes mellitus |
| E119 | Noninsulindependent diabetes mellitus without complications | diabetes mellitus |
| E120 | Malnutritionrelated diabetes mellitus with coma | diabetes mellitus |
| E121 | Malnutritionrelated diabetes mellitus with ketoacidosis | diabetes mellitus |
| E122 | Malnutritionrelated diabetes mellitus with renal complications | diabetes mellitus |
| E123 | Malnutritionrelated diabetes mellitus with ophthalmic complications | diabetes mellitus |
| E124 | Malnutritionrelated diabetes mellitus with neurological complications | diabetes mellitus |
| E125 | Malnutritionrelated diabetes mellitus with peripheral circulatory complications | diabetes mellitus |
| E126 | Malnutritionrelated diabetes mellitus with other specified complications | diabetes mellitus |
| E127 | Malnutritionrelated diabetes mellitus with multiple complications | diabetes mellitus |
| E128 | Malnutritionrelated diabetes mellitus with unspecified complications | diabetes mellitus |
| E129 | Malnutritionrelated diabetes mellitus without complications | diabetes mellitus |
| E130 | Other specified diabetes mellitus with coma | diabetes mellitus |
| E131 | Other specified diabetes mellitus with ketoacidosis | diabetes mellitus |
| E132 | Other specified diabetes mellitus with renal complications | diabetes mellitus |
| E133 | Other specified diabetes mellitus with ophthalmic complications | diabetes mellitus |
| E134 | Other specified diabetes mellitus with neurological complications | diabetes mellitus |
| E135 | Other specified diabetes mellitus with peripheral circulatory complications | diabetes mellitus |
| E136 | Other specified diabetes mellitus with other specified complications | diabetes mellitus |
| E137 | Other specified diabetes mellitus with multiple complications | diabetes mellitus |
| E138 | Other specified diabetes mellitus with unspecified complications | diabetes mellitus |
| E139 | Other specified diabetes mellitus without complications | diabetes mellitus |
| E140 | Unspecified diabetes mellitus with coma | diabetes mellitus |
| E141 | Unspecified diabetes mellitus with ketoacidosis | diabetes mellitus |
| E142 | Unspecified diabetes mellitus with renal complications | diabetes mellitus |
| E143 | Unspecified diabetes mellitus with ophthalmic complications | diabetes mellitus |
| E144 | Unspecified diabetes mellitus with neurological complications | diabetes mellitus |
| E145 | Unspecified diabetes mellitus with peripheral circulatory complications | diabetes mellitus |
| E146 | Unspecified diabetes mellitus with other specified complications | diabetes mellitus |
| E147 | Unspecified diabetes mellitus with multiple complications | diabetes mellitus |
| E148 | Unspecified diabetes mellitus with unspecified complications | diabetes mellitus |
| E149 | Unspecified diabetes mellitus without complications | diabetes mellitus |
| O240 | Preexisting diabetes mellitus insulindependent | diabetes mellitus |
| O241 | Preexisting diabetes mellitus noninsulindependent | diabetes mellitus |
| O242 | Preexisting malnutritionrelated diabetes mellitus | diabetes mellitus |
| O243 | Preexisting diabetes mellitus unspecified | diabetes mellitus |
| O244 | Diabetes mellitus arising in pregnancy | diabetes mellitus |
| O249 | Diabetes mellitus in pregnancy unspecified | diabetes mellitus |
| K721 | Chronic hepatic failure | hepatic |
| K730 | Chronic persistent hepatitis not elsewhere classified | hepatic |
| K731 | Chronic lobular hepatitis not elsewhere classified | hepatic |
| K732 | Chronic active hepatitis not elsewhere classified | hepatic |
| K738 | Other chronic hepatitis not elsewhere classified | hepatic |
| K739 | Chronic hepatitis unspecified | hepatic |
| K740 | Hepatic fibrosis | hepatic |
| K741 | Hepatic sclerosis | hepatic |
| K742 | Hepatic fibrosis with hepatic sclerosis | hepatic |
| K743 | Primary biliary cirrhosis | hepatic |
| K744 | Secondary biliary cirrhosis | hepatic |
| K745 | Biliary cirrhosis unspecified | hepatic |
| K746 | Other and unspecified cirrhosis of liver | hepatic |
| K763 | Infarction of liver | hepatic |
| K767 | Hepatorenal syndrome | hepatic |
| K700 | Alcoholic fatty liver | hepatic/alcohol |
| K701 | Alcoholic hepatitis | hepatic/alcohol |
| K702 | Alcoholic fibrosis and sclerosis of liver | hepatic/alcohol |
| K703 | Alcoholic cirrhosis of liver | hepatic/alcohol |
| K704 | Alcoholic hepatic failure | hepatic/alcohol |
| B200 | HIV disease resulting in mycobacterial infection | HIV |
| B201 | HIV disease resulting in other bacterial infections | HIV |
| B202 | HIV disease resulting in cytomegaloviral disease | HIV |
| B203 | HIV disease resulting in other viral infections | HIV |
| B204 | HIV disease resulting in candidiasis | HIV |
| B205 | HIV disease resulting in other mycoses | HIV |
| B206 | HIV disease resulting in Pneumocystis carinii pneumonia | HIV |
| B207 | HIV disease resulting in multiple infections | HIV |
| B208 | HIV disease resulting in other infectious and parasitic diseases | HIV |
| B209 | HIV disease resulting in unspecified infectious or parasitic disease | HIV |
| B210 | HIV disease resulting in Kaposi's sarcoma | HIV |
| B211 | HIV disease resulting in Burkitt's lymphoma | HIV |
| B212 | HIV disease resulting in other types of nonHodgkin's lymphoma | HIV |
| B213 | HIV disease resulting in other malignant neoplasms of lymphoid haematopoietic and related tissue | HIV |
| B217 | HIV disease resulting in multiple malignant neoplasms | HIV |
| B218 | HIV disease resulting in other malignant neoplasms | HIV |
| B219 | HIV disease resulting in unspecified malignant neoplasm | HIV |
| B220 | HIV disease resulting in encephalopathy | HIV |
| B221 | HIV disease resulting in lymphoid interstitial pneumonitis | HIV |
| B222 | HIV disease resulting in wasting syndrome | HIV |
| B227 | HIV disease resulting in multiple diseases classified elsewhere | HIV |
| B230 | Acute HIV infection syndrome | HIV |
| B231 | HIV disease resulting in (persistent) generalized lymphadenopathy | HIV |
| B232 | HIV disease resulting in haematological and immunological abnormalities not elsewhere classified | HIV |
| B238 | HIV disease resulting in other specified conditions | HIV |
| B24 | Unspecified human immunodeficiency virus [HIV] disease | HIV |
| R75 | Laboratory evidence of human immunodeficiency virus [HIV] | HIV |
| I10 | Essential (primary) hypertension | hypertension |
| I110 | Hypertensive heart disease with (congestive) heart failure | hypertension |
| I119 | Hypertensive heart disease without (congestive) heart failure | hypertension |
| I120 | Hypertensive renal disease with renal failure | hypertension |
| I129 | Hypertensive renal disease without renal failure | hypertension |
| I130 | Hypertensive heart and renal disease with (congestive) heart failure | hypertension |
| I131 | Hypertensive heart and renal disease with renal failure | hypertension |
| I132 | Hypertensive heart and renal disease with both (congestive) heart failure and renal failure | hypertension |
| I139 | Hypertensive heart and renal disease unspecified | hypertension |
| I150 | Renovascular hypertension | hypertension |
| I151 | Hypertension secondary to other renal disorders | hypertension |
| I152 | Hypertension secondary to endocrine disorders | hypertension |
| I158 | Other secondary hypertension | hypertension |
| I159 | Secondary hypertension unspecified | hypertension |
| N180 | Endstage renal disease | renal |
| N188 | Other chronic renal failure | renal |
| N189 | Chronic renal failure unspecified | renal |

Legend: COPD = chronic obstructive pulmonar disease; HIV = human immunodeficiency virus

Table S4. International Classification of Diseases 10th Revision (ICD-10) codes used for infection definition (bacterial, protozoan, viral or fungal).

| **ICD-10 code** | **Description** |
| --- | --- |
| A000 | Cholera due to Vibrio cholerae 01 biovar cholerae |
| A001 | Cholera due to Vibrio cholerae 01 biovar eltor |
| A009 | Cholera unspecified |
| A010 | Typhoid fever |
| A011 | Paratyphoid fever A |
| A012 | Paratyphoid fever B |
| A013 | Paratyphoid fever C |
| A014 | Paratyphoid fever unspecified |
| A020 | Salmonella enteritis |
| A021 | Salmonella septicaemia |
| A022 | Localized salmonella infections |
| A028 | Other specified salmonella infections |
| A029 | Salmonella infection unspecified |
| A030 | Shigellosis due to Shigella dysenteriae |
| A031 | Shigellosis due to Shigella flexneri |
| A032 | Shigellosis due to Shigella boydii |
| A033 | Shigellosis due to Shigella sonnei |
| A038 | Other shigellosis |
| A039 | Shigellosis unspecified |
| A040 | Enteropathogenic Escherichia coli infection |
| A041 | Enterotoxigenic Escherichia coli infection |
| A042 | Enteroinvasive Escherichia coli infection |
| A043 | Enterohaemorrhagic Escherichia coli infection |
| A044 | Other intestinal Escherichia coli infections |
| A045 | Campylobacter enteritis |
| A046 | Enteritis due to Yersinia enterocolitica |
| A047 | Enterocolitis due to Clostridium difficile |
| A048 | Other specified bacterial intestinal infections |
| A049 | Bacterial intestinal infection unspecified |
| A050 | Foodborne staphylococcal intoxication |
| A051 | Botulism |
| A052 | Foodborne Clostridium perfringens [Clostridium welchii] intoxication |
| A053 | Foodborne Vibrio parahaemolyticus intoxication |
| A054 | Foodborne Bacillus cereus intoxication |
| A058 | Other specified bacterial foodborne intoxications |
| A059 | Bacterial foodborne intoxication unspecified |
| A060 | Acute amoebic dysentery |
| A061 | Chronic intestinal amoebiasis |
| A062 | Amoebic nondysenteric colitis |
| A063 | Amoeboma of intestine |
| A064 | Amoebic liver abscess |
| A065 | Amoebic lung abscess |
| A066 | Amoebic brain abscess |
| A067 | Cutaneous amoebiasis |
| A068 | Amoebic infection of other sites |
| A069 | Amoebiasis unspecified |
| A070 | Balantidiasis |
| A071 | Giardiasis [lambliasis] |
| A072 | Cryptosporidiosis |
| A073 | Isosporiasis |
| A078 | Other specified protozoal intestinal diseases |
| A079 | Protozoal intestinal disease unspecified |
| A080 | Rotaviral enteritis |
| A081 | Acute gastroenteropathy due to Norwalk agent |
| A082 | Adenoviral enteritis |
| A083 | Other viral enteritis |
| A084 | Viral intestinal infection unspecified |
| A085 | Other specified intestinal infections |
| A09 | Diarrhoea and gastroenteritis of presumed infectious origin |
| A150 | Tuberculosis of lung confirmed by sputum microscopy with or without culture |
| A151 | Tuberculosis of lung confirmed by culture only |
| A152 | Tuberculosis of lung confirmed histologically |
| A153 | Tuberculosis of lung confirmed by unspecified means |
| A154 | Tuberculosis of intrathoracic lymph nodes confirmed bacteriologically and histologically |
| A155 | Tuberculosis of larynx trachea and bronchus confirmed bacteriologically and histologically |
| A156 | Tuberculous pleurisy confirmed bacteriologically and histologically |
| A157 | Primary respiratory tuberculosis confirmed bacteriologically and histologically |
| A158 | Other respiratory tuberculosis confirmed bacteriologically and histologically |
| A159 | Respiratory tuberculosis unspecified confirmed bacteriologically and histologically |
| A160 | Tuberculosis of lung bacteriologically and histologically negative |
| A161 | Tuberculosis of lung bacteriological and histological examination not done |
| A162 | Tuberculosis of lung without mention of bacteriological or histological confirmation |
| A163 | Tuberculosis of intrathoracic lymph nodes without mention of bacteriological or histological confirmation |
| A164 | Tuberculosis of larynx trachea and bronchus without mention of bacteriological or histological confirmation |
| A165 | Tuberculous pleurisy without mention of bacteriological or histological confirmation |
| A167 | Primary respiratory tuberculosis without mention of bacteriological or histological confirmation |
| A168 | Other respiratory tuberculosis without mention of bacteriological or histological confirmation |
| A169 | Respiratory tuberculosis unspecified without mention of bacteriological or histological confirmation |
| A170 | Tuberculous meningitis |
| A171 | Meningeal tuberculoma |
| A178 | Other tuberculosis of nervous system |
| A179 | Tuberculosis of nervous system unspecified |
| A180 | Tuberculosis of bones and joints |
| A181 | Tuberculosis of genitourinary system |
| A182 | Tuberculous peripheral lymphadenopathy |
| A183 | Tuberculosis of intestines peritoneum and mesenteric glands |
| A187 | Tuberculosis of adrenal glands |
| A190 | Acute miliary tuberculosis of a single specified site |
| A191 | Acute miliary tuberculosis of multiple sites |
| A192 | Acute miliary tuberculosis unspecified |
| A198 | Other miliary tuberculosis |
| A199 | Miliary tuberculosis unspecified |
| A200 | Bubonic plague |
| A201 | Cellulocutaneous plague |
| A202 | Pneumonic plague |
| A203 | Plague meningitis |
| A207 | Septicaemic plague |
| A208 | Other forms of plague |
| A209 | Plague unspecified |
| A210 | Ulceroglandular tularaemia |
| A211 | Oculoglandular tularaemia |
| A212 | Pulmonary tularaemia |
| A213 | Gastrointestinal tularaemia |
| A217 | Generalized tularaemia |
| A218 | Other forms of tularaemia |
| A219 | Tularaemia unspecified |
| A220 | Cutaneous anthrax |
| A221 | Pulmonary anthrax |
| A222 | Gastrointestinal anthrax |
| A227 | Anthrax septicaemia |
| A228 | Other forms of anthrax |
| A229 | Anthrax unspecified |
| A230 | Brucellosis due to Brucella melitensis |
| A231 | Brucellosis due to Brucella abortus |
| A232 | Brucellosis due to Brucella suis |
| A233 | Brucellosis due to Brucella canis |
| A238 | Other brucellosis |
| A239 | Brucellosis unspecified |
| A250 | Spirillosis |
| A251 | Streptobacillosis |
| A259 | Ratbite fever unspecified |
| A267 | Erysipelothrix septicaemia |
| A268 | Other forms of erysipeloid |
| A270 | Leptospirosis icterohaemorrhagica |
| A278 | Other forms of leptospirosis |
| A279 | Leptospirosis unspecified |
| A310 | Pulmonary mycobacterial infection |
| A311 | Cutaneous mycobacterial infection |
| A318 | Other mycobacterial infections |
| A319 | Mycobacterial infection unspecified |
| A320 | Cutaneous listeriosis |
| A321 | Listerial meningitis and meningoencephalitis |
| A327 | Listerial septicaemia |
| A328 | Other forms of listeriosis |
| A329 | Listeriosis unspecified |
| A33 | Tetanus neonatorum |
| A34 | Obstetrical tetanus |
| A35 | Other tetanus |
| A360 | Pharyngeal diphtheria |
| A361 | Nasopharyngeal diphtheria |
| A362 | Laryngeal diphtheria |
| A363 | Cutaneous diphtheria |
| A368 | Other diphtheria |
| A369 | Diphtheria unspecified |
| A370 | Whooping cough due to Bordetella pertussis |
| A371 | Whooping cough due to Bordetella parapertussis |
| A378 | Whooping cough due to other Bordetella species |
| A379 | Whooping cough unspecified |
| A38 | Scarlet fever |
| A390 | Meningococcal meningitis |
| A391 | WaterhouseFriderichsen syndrome |
| A392 | Acute meningococcaemia |
| A393 | Chronic meningococcaemia |
| A394 | Meningococcaemia unspecified |
| A395 | Meningococcal heart disease |
| A398 | Other meningococcal infections |
| A399 | Meningococcal infection unspecified |
| A400 | Septicaemia due to streptococcus group A |
| A401 | Septicaemia due to streptococcus group B |
| A402 | Septicaemia due to streptococcus group D |
| A403 | Septicaemia due to Streptococcus pneumoniae |
| A408 | Other streptococcal septicaemia |
| A409 | Streptococcal septicaemia unspecified |
| A410 | Septicaemia due to Staphylococcus aureus |
| A411 | Septicaemia due to other specified staphylococcus |
| A412 | Septicaemia due to unspecified staphylococcus |
| A413 | Septicaemia due to Haemophilus influenzae |
| A414 | Septicaemia due to anaerobes |
| A415 | Septicaemia due to other Gramnegative organisms |
| A418 | Other specified septicaemia |
| A419 | Septicaemia unspecified |
| A420 | Pulmonary actinomycosis |
| A421 | Abdominal actinomycosis |
| A422 | Cervicofacial actinomycosis |
| A427 | Actinomycotic septicaemia |
| A428 | Other forms of actinomycosis |
| A429 | Actinomycosis unspecified |
| A430 | Pulmonary nocardiosis |
| A431 | Cutaneous nocardiosis |
| A438 | Other forms of nocardiosis |
| A439 | Nocardiosis unspecified |
| A440 | Systemic bartonellosis |
| A441 | Cutaneous and mucocutaneous bartonellosis |
| A448 | Other forms of bartonellosis |
| A449 | Bartonellosis unspecified |
| A46 | Erysipelas |
| A480 | Gas gangrene |
| A481 | Legionnaires' disease |
| A482 | Nonpneumonic Legionnaires' disease [Pontiac fever] |
| A483 | Toxic shock syndrome |
| A484 | Brazilian purpuric fever |
| A488 | Other specified bacterial diseases |
| A490 | Staphylococcal infection unspecified |
| A491 | Streptococcal infection unspecified |
| A492 | Haemophilus influenzae infection unspecified |
| A493 | Mycoplasma infection unspecified |
| A498 | Other bacterial infections of unspecified site |
| A499 | Bacterial infection unspecified |
| A500 | Early congenital syphilis symptomatic |
| A501 | Early congenital syphilis latent |
| A502 | Early congenital syphilis unspecified |
| A503 | Late congenital syphilitic oculopathy |
| A504 | Late congenital neurosyphilis [juvenile neurosyphilis] |
| A505 | Other late congenital syphilis symptomatic |
| A506 | Late congenital syphilis latent |
| A507 | Late congenital syphilis unspecified |
| A509 | Congenital syphilis unspecified |
| A510 | Primary genital syphilis |
| A511 | Primary anal syphilis |
| A512 | Primary syphilis of other sites |
| A513 | Secondary syphilis of skin and mucous membranes |
| A514 | Other secondary syphilis |
| A515 | Early syphilis latent |
| A519 | Early syphilis unspecified |
| A530 | Latent syphilis unspecified as early or late |
| A539 | Syphilis unspecified |
| A690 | Necrotizing ulcerative stomatitis |
| A691 | Other Vincent's infections |
| A692 | Lyme disease |
| A698 | Other specified spirochaetal infections |
| A699 | Spirochaetal infection unspecified |
| A70 | Chlamydia psittaci infection |
| A740 | Chlamydial conjunctivitis |
| A748 | Other chlamydial diseases |
| A749 | Chlamydial infection unspecified |
| A750 | Epidemic louseborne typhus fever due to Rickettsia prowazekii |
| A751 | Recrudescent typhus [Brill's disease] |
| A752 | Typhus fever due to Rickettsia typhi |
| A753 | Typhus fever due to Rickettsia tsutsugamushi |
| A759 | Typhus fever unspecified |
| A770 | Spotted fever due to Rickettsia rickettsii |
| A771 | Spotted fever due to Rickettsia conorii |
| A772 | Spotted fever due to Rickettsia sibirica |
| A773 | Spotted fever due to Rickettsia australis |
| A778 | Other spotted fevers |
| A779 | Spotted fever unspecified |
| A790 | Trench fever |
| A791 | Rickettsialpox due to Rickettsia akari |
| A798 | Other specified rickettsioses |
| A799 | Rickettsiosis unspecified |
| A800 | Acute paralytic poliomyelitis vaccineassociated |
| A801 | Acute paralytic poliomyelitis wild virus imported |
| A802 | Acute paralytic poliomyelitis wild virus indigenous |
| A803 | Acute paralytic poliomyelitis other and unspecified |
| A804 | Acute nonparalytic poliomyelitis |
| A809 | Acute poliomyelitis unspecified |
| A818 | Other atypical virus infections of central nervous system |
| A819 | Atypical virus infection of central nervous system unspecified |
| A820 | Sylvatic rabies |
| A821 | Urban rabies |
| A829 | Rabies unspecified |
| A830 | Japanese encephalitis |
| A831 | Western equine encephalitis |
| A832 | Eastern equine encephalitis |
| A833 | St Louis encephalitis |
| A834 | Australian encephalitis |
| A835 | California encephalitis |
| A836 | Rocio virus disease |
| A838 | Other mosquitoborne viral encephalitis |
| A839 | Mosquitoborne viral encephalitis unspecified |
| A840 | Far Eastern tickborne encephalitis [Russian springsummer encephalitis] |
| A841 | Central European tickborne encephalitis |
| A848 | Other tickborne viral encephalitis |
| A849 | Tickborne viral encephalitis unspecified |
| A850 | Enteroviral encephalitis |
| A851 | Adenoviral encephalitis |
| A852 | Arthropodborne viral encephalitis unspecified |
| A858 | Other specified viral encephalitis |
| A86 | Unspecified viral encephalitis |
| A870 | Enteroviral meningitis |
| A871 | Adenoviral meningitis |
| A872 | Lymphocytic choriomeningitis |
| A878 | Other viral meningitis |
| A879 | Viral meningitis unspecified |
| A880 | Enteroviral exanthematous fever [Boston exanthem] |
| A881 | Epidemic vertigo |
| A888 | Other specified viral infections of central nervous system |
| A89 | Unspecified viral infection of central nervous system |
| A90 | Dengue fever [classical dengue] |
| A91 | Dengue haemorrhagic fever |
| A920 | Chikungunya virus disease |
| A921 | O'nyongnyong fever |
| A922 | Venezuelan equine fever |
| A923 | West Nile fever |
| A924 | Rift Valley fever |
| A928 | Other specified mosquitoborne viral fevers |
| A929 | Mosquitoborne viral fever unspecified |
| A930 | Oropouche virus disease |
| A931 | Sandfly fever |
| A932 | Colorado tick fever |
| A938 | Other specified arthropodborne viral fevers |
| A94 | Unspecified arthropodborne viral fever |
| A950 | Sylvatic yellow fever |
| A951 | Urban yellow fever |
| A959 | Yellow fever unspecified |
| A960 | Junin haemorrhagic fever |
| A961 | Machupo haemorrhagic fever |
| A962 | Lassa fever |
| A968 | Other arenaviral haemorrhagic fevers |
| A969 | Arenaviral haemorrhagic fever unspecified |
| A980 | CrimeanCongo haemorrhagic fever |
| A981 | Omsk haemorrhagic fever |
| A982 | Kyasanur Forest disease |
| A983 | Marburg virus disease |
| A984 | Ebola virus disease |
| A985 | Haemorrhagic fever with renal syndrome |
| A988 | Other specified viral haemorrhagic fevers |
| A99 | Unspecified viral haemorrhagic fever |
| AAA | All causes |
| B002 | Herpesviral gingivostomatitis and pharyngotonsillitis |
| B003 | Herpesviral meningitis |
| B004 | Herpesviral encephalitis |
| B005 | Herpesviral ocular disease |
| B007 | Disseminated herpesviral disease |
| B008 | Other forms of herpesviral infection |
| B009 | Herpesviral infection unspecified |
| B010 | Varicella meningitis |
| B011 | Varicella encephalitis |
| B012 | Varicella pneumonia |
| B018 | Varicella with other complications |
| B150 | Hepatitis A with hepatic coma |
| B159 | Hepatitis A without hepatic coma |
| B160 | Acute hepatitis B with deltaagent (coinfection) with hepatic coma |
| B161 | Acute hepatitis B with deltaagent (coinfection) without hepatic coma |
| B162 | Acute hepatitis B without deltaagent with hepatic coma |
| B169 | Acute hepatitis B without deltaagent and without hepatic coma |
| B170 | Acute delta(super)infection of hepatitis B carrier |
| B171 | Acute hepatitis C |
| B172 | Acute hepatitis E |
| B178 | Other specified acute viral hepatitis |
| B200 | HIV disease resulting in mycobacterial infection |
| B201 | HIV disease resulting in other bacterial infections |
| B202 | HIV disease resulting in cytomegaloviral disease |
| B203 | HIV disease resulting in other viral infections |
| B204 | HIV disease resulting in candidiasis |
| B205 | HIV disease resulting in other mycoses |
| B206 | HIV disease resulting in Pneumocystis carinii pneumonia |
| B207 | HIV disease resulting in multiple infections |
| B208 | HIV disease resulting in other infectious and parasitic diseases |
| B209 | HIV disease resulting in unspecified infectious or parasitic disease |
| B250 | Cytomegaloviral pneumonitis |
| B251 | Cytomegaloviral hepatitis |
| B252 | Cytomegaloviral pancreatitis |
| B258 | Other cytomegaloviral diseases |
| B259 | Cytomegaloviral disease unspecified |
| B270 | Gammaherpesviral mononucleosis |
| B271 | Cytomegaloviral mononucleosis |
| B278 | Other infectious mononucleosis |
| B279 | Infectious mononucleosis unspecified |
| B370 | Candidal stomatitis |
| B371 | Pulmonary candidiasis |
| B373 | Candidiasis of vulva and vagina |
| B374 | Candidiasis of other urogenital sites |
| B375 | Candidal meningitis |
| B376 | Candidal endocarditis |
| B377 | Candidal septicaemia |
| B378 | Candidiasis of other sites |
| B379 | Candidiasis unspecified |
| B380 | Acute pulmonary coccidioidomycosis |
| B381 | Chronic pulmonary coccidioidomycosis |
| B382 | Pulmonary coccidioidomycosis unspecified |
| B383 | Cutaneous coccidioidomycosis |
| B384 | Coccidioidomycosis meningitis |
| B387 | Disseminated coccidioidomycosis |
| B388 | Other forms of coccidioidomycosis |
| B389 | Coccidioidomycosis unspecified |
| B390 | Acute pulmonary histoplasmosis capsulati |
| B391 | Chronic pulmonary histoplasmosis capsulati |
| B392 | Pulmonary histoplasmosis capsulati unspecified |
| B393 | Disseminated histoplasmosis capsulati |
| B394 | Histoplasmosis capsulati unspecified |
| B395 | Histoplasmosis duboisii |
| B399 | Histoplasmosis unspecified |
| B400 | Acute pulmonary blastomycosis |
| B401 | Chronic pulmonary blastomycosis |
| B402 | Pulmonary blastomycosis unspecified |
| B403 | Cutaneous blastomycosis |
| B407 | Disseminated blastomycosis |
| B408 | Other forms of blastomycosis |
| B409 | Blastomycosis unspecified |
| B410 | Pulmonary paracoccidioidomycosis |
| B417 | Disseminated paracoccidioidomycosis |
| B418 | Other forms of paracoccidioidomycosis |
| B419 | Paracoccidioidomycosis unspecified |
| B440 | Invasive pulmonary aspergillosis |
| B441 | Other pulmonary aspergillosis |
| B442 | Tonsillar aspergillosis |
| B447 | Disseminated aspergillosis |
| B448 | Other forms of aspergillosis |
| B449 | Aspergillosis unspecified |
| B450 | Pulmonary cryptococcosis |
| B451 | Cerebral cryptococcosis |
| B453 | Osseous cryptococcosis |
| B457 | Disseminated cryptococcosis |
| B458 | Other forms of cryptococcosis |
| B459 | Cryptococcosis unspecified |
| B500 | Plasmodium falciparum malaria with cerebral complications |
| B508 | Other severe and complicated Plasmodium falciparum malaria |
| B509 | Plasmodium falciparum malaria unspecified |
| B510 | Plasmodium vivax malaria with rupture of spleen |
| B518 | Plasmodium vivax malaria with other complications |
| B519 | Plasmodium vivax malaria without complication |
| B520 | Plasmodium malariae malaria with nephropathy |
| B528 | Plasmodium malariae malaria with other complications |
| B529 | Plasmodium malariae malaria without complication |
| B530 | Plasmodium ovale malaria |
| B531 | Malaria due to simian plasmodia |
| B538 | Other parasitologically confirmed malaria not elsewhere classified |
| B54 | Unspecified malaria |
| B550 | Visceral leishmaniasis |
| B551 | Cutaneous leishmaniasis |
| B552 | Mucocutaneous leishmaniasis |
| B559 | Leishmaniasis unspecified |
| B580 | Toxoplasma oculopathy |
| B581 | Toxoplasma hepatitis |
| B582 | Toxoplasma meningoencephalitis |
| B583 | Pulmonary toxoplasmosis |
| B588 | Toxoplasmosis with other organ involvement |
| B589 | Toxoplasmosis unspecified |
| B59 | Pneumocystosis |
| B600 | Babesiosis |
| B601 | Acanthamoebiasis |
| B602 | Naegleriasis |
| B608 | Other specified protozoal diseases |
| B64 | Unspecified protozoal disease |
| B780 | Intestinal strongyloidiasis |
| B781 | Cutaneous strongyloidiasis |
| B787 | Disseminated strongyloidiasis |
| B789 | Strongyloidiasis unspecified |
| G000 | Haemophilus meningitis |
| G001 | Pneumococcal meningitis |
| G002 | Streptococcal meningitis |
| G003 | Staphylococcal meningitis |
| G008 | Other bacterial meningitis |
| G009 | Bacterial meningitis unspecified |
| G030 | Nonpyogenic meningitis |
| G031 | Chronic meningitis |
| G032 | Benign recurrent meningitis [Mollaret] |
| G038 | Meningitis due to other specified causes |
| G039 | Meningitis unspecified |
| G040 | Acute disseminated encephalitis |
| G041 | Tropical spastic paraplegia |
| G042 | Bacterial meningoencephalitis and meningomyelitis not elsewhere classified |
| G048 | Other encephalitis myelitis and encephalomyelitis |
| G049 | Encephalitis myelitis and encephalomyelitis unspecified |
| G060 | Intracranial abscess and granuloma |
| G061 | Intraspinal abscess and granuloma |
| G062 | Extradural and subdural abscess unspecified |
| H650 | Acute serous otitis media |
| H651 | Other acute nonsuppurative otitis media |
| H652 | Chronic serous otitis media |
| H653 | Chronic mucoid otitis media |
| H654 | Other chronic nonsuppurative otitis media |
| H659 | Nonsuppurative otitis media unspecified |
| H660 | Acute suppurative otitis media |
| H661 | Chronic tubotympanic suppurative otitis media |
| H662 | Chronic atticoantral suppurative otitis media |
| H663 | Other chronic suppurative otitis media |
| H664 | Suppurative otitis media unspecified |
| H669 | Otitis media unspecified |
| H700 | Acute mastoiditis |
| H701 | Chronic mastoiditis |
| H702 | Petrositis |
| H708 | Other mastoiditis and related conditions |
| H709 | Mastoiditis unspecified |
| I301 | Infective pericarditis |
| I308 | Other forms of acute pericarditis |
| I309 | Acute pericarditis unspecified |
| I330 | Acute and subacute infective endocarditis |
| I339 | Acute endocarditis unspecified |
| I38 | Endocarditis valve unspecified |
| I400 | Infective myocarditis |
| J00 | Acute nasopharyngitis [common cold] |
| J010 | Acute maxillary sinusitis |
| J011 | Acute frontal sinusitis |
| J012 | Acute ethmoidal sinusitis |
| J013 | Acute sphenoidal sinusitis |
| J014 | Acute pansinusitis |
| J018 | Other acute sinusitis |
| J019 | Acute sinusitis unspecified |
| J020 | Streptococcal pharyngitis |
| J028 | Acute pharyngitis due to other specified organisms |
| J029 | Acute pharyngitis unspecified |
| J030 | Streptococcal tonsillitis |
| J038 | Acute tonsillitis due to other specified organisms |
| J039 | Acute tonsillitis unspecified |
| J040 | Acute laryngitis |
| J041 | Acute tracheitis |
| J042 | Acute laryngotracheitis |
| J050 | Acute obstructive laryngitis [croup] |
| J051 | Acute epiglottitis |
| J060 | Acute laryngopharyngitis |
| J068 | Other acute upper respiratory infections of multiple sites |
| J069 | Acute upper respiratory infection unspecified |
| J09 | Influenza due to identified avian influenza virus |
| J100 | Influenza with pneumonia influenza virus identified |
| J101 | Influenza with other respiratory manifestations influenza virus identified |
| J108 | Influenza with other manifestations influenza virus identified |
| J110 | Influenza with pneumonia virus not identified |
| J111 | Influenza with other respiratory manifestations virus not identified |
| J118 | Influenza with other manifestations virus not identified |
| J120 | Adenoviral pneumonia |
| J121 | Respiratory syncytial virus pneumonia |
| J122 | Parainfluenza virus pneumonia |
| J128 | Other viral pneumonia |
| J129 | Viral pneumonia unspecified |
| J13 | Pneumonia due to Streptococcus pneumoniae |
| J14 | Pneumonia due to Haemophilus influenzae |
| J150 | Pneumonia due to Klebsiella pneumoniae |
| J151 | Pneumonia due to Pseudomonas |
| J152 | Pneumonia due to staphylococcus |
| J153 | Pneumonia due to streptococcus group B |
| J154 | Pneumonia due to other streptococci |
| J155 | Pneumonia due to Escherichia coli |
| J156 | Pneumonia due to other aerobic Gramnegative bacteria |
| J157 | Pneumonia due to Mycoplasma pneumoniae |
| J158 | Other bacterial pneumonia |
| J159 | Bacterial pneumonia unspecified |
| J160 | Chlamydial pneumonia |
| J168 | Pneumonia due to other specified infectious organisms |
| J180 | Bronchopneumonia unspecified |
| J181 | Lobar pneumonia unspecified |
| J182 | Hypostatic pneumonia unspecified |
| J188 | Other pneumonia organism unspecified |
| J189 | Pneumonia unspecified |
| J200 | Acute bronchitis due to Mycoplasma pneumoniae |
| J201 | Acute bronchitis due to Haemophilus influenzae |
| J202 | Acute bronchitis due to streptococcus |
| J203 | Acute bronchitis due to coxsackievirus |
| J204 | Acute bronchitis due to parainfluenza virus |
| J205 | Acute bronchitis due to respiratory syncytial virus |
| J206 | Acute bronchitis due to rhinovirus |
| J207 | Acute bronchitis due to echovirus |
| J208 | Acute bronchitis due to other specified organisms |
| J209 | Acute bronchitis unspecified |
| J210 | Acute bronchiolitis due to respiratory syncytial virus |
| J218 | Acute bronchiolitis due to other specified organisms |
| J219 | Acute bronchiolitis unspecified |
| J22 | Unspecified acute lower respiratory infection |
| J36 | Peritonsillar abscess |
| J390 | Retropharyngeal and parapharyngeal abscess |
| J391 | Other abscess of pharynx |
| J40 | Bronchitis not specified as acute or chronic |
| J851 | Abscess of lung with pneumonia |
| J852 | Abscess of lung without pneumonia |
| J853 | Abscess of mediastinum |
| J860 | Pyothorax with fistula |
| J869 | Pyothorax without fistula |
| K102 | Inflammatory conditions of jaws |
| K113 | Abscess of salivary gland |
| K122 | Cellulitis and abscess of mouth |
| K350 | Acute appendicitis with generalized peritonitis |
| K351 | Acute appendicitis with peritoneal absess |
| K359 | Acute appendicitis unspecified |
| K36 | Other appendicitis |
| K37 | Unspecified appendicitis |
| K570 | Diverticular disease of small intestine with perforation and abscess |
| K571 | Diverticular disease of small intestine without perforation or abscess |
| K572 | Diverticular disease of large intestine with perforation and abscess |
| K573 | Diverticular disease of large intestine without perforation or abscess |
| K574 | Diverticular disease of both small and large intestine with perforation and abscess |
| K575 | Diverticular disease of both small and large intestine without perforation or abscess |
| K578 | Diverticular disease of intestine part unspecified with perforation and abscess |
| K579 | Diverticular disease of intestine part unspecified without perforation or abscess |
| K610 | Anal abscess |
| K611 | Rectal abscess |
| K612 | Anorectal abscess |
| K613 | Ischiorectal abscess |
| K614 | Intrasphincteric abscess |
| K630 | Abscess of intestine |
| K650 | Acute peritonitis |
| K658 | Other peritonitis |
| K659 | Peritonitis unspecified |
| K750 | Abscess of liver |
| K800 | Calculus of gallbladder with acute cholecystitis |
| K801 | Calculus of gallbladder with other cholecystitis |
| K802 | Calculus of gallbladder without cholecystitis |
| K803 | Calculus of bile duct with cholangitis |
| K804 | Calculus of bile duct with cholecystitis |
| K810 | Acute cholecystitis |
| K818 | Other cholecystitis |
| K830 | Cholangitis |
| K850 | Idiopathic acute pancreatitis |
| K851 | Biliary acute pancreatitis |
| K852 | Alcoholinduced acute pancreatitis |
| K853 | Druginduced acute pancreatitis |
| K858 | Other acute pancreatitis |
| K859 | Acute pancreatitis unspecified |
| L00 | Staphylococcal scalded skin syndrome |
| L010 | Impetigo [any organism] [any site] |
| L011 | Impetiginization of other dermatoses |
| L020 | Cutaneous abscess furuncle and carbuncle of face |
| L021 | Cutaneous abscess furuncle and carbuncle of neck |
| L022 | Cutaneous abscess furuncle and carbuncle of trunk |
| L023 | Cutaneous abscess furuncle and carbuncle of buttock |
| L024 | Cutaneous abscess furuncle and carbuncle of limb |
| L028 | Cutaneous abscess furuncle and carbuncle of other sites |
| L029 | Cutaneous abscess furuncle and carbuncle unspecified |
| L030 | Cellulitis of finger and toe |
| L031 | Cellulitis of other parts of limb |
| L032 | Cellulitis of face |
| L033 | Cellulitis of trunk |
| L038 | Cellulitis of other sites |
| L039 | Cellulitis unspecified |
| L040 | Acute lymphadenitis of face head and neck |
| L041 | Acute lymphadenitis of trunk |
| L042 | Acute lymphadenitis of upper limb |
| L043 | Acute lymphadenitis of lower limb |
| L048 | Acute lymphadenitis of other sites |
| L049 | Acute lymphadenitis unspecified |
| L050 | Pilonidal cyst with abscess |
| L059 | Pilonidal cyst without abscess |
| L080 | Pyoderma |
| L081 | Erythrasma |
| L088 | Other specified local infections of skin and subcutaneous tissue |
| L089 | Local infection of skin and subcutaneous tissue unspecified |
| M600 | Infective myositis |
| M601 | Interstitial myositis |
| M860 | Acute haematogenous osteomyelitis |
| M861 | Other acute osteomyelitis |
| M862 | Subacute osteomyelitis |
| M863 | Chronic multifocal osteomyelitis |
| M864 | Chronic osteomyelitis with draining sinus |
| M865 | Other chronic haematogenous osteomyelitis |
| M866 | Other chronic osteomyelitis |
| M868 | Other osteomyelitis |
| M869 | Osteomyelitis unspecified |
| N151 | Renal and perinephric abscess |
| N300 | Acute cystitis |
| N308 | Other cystitis |
| N309 | Cystitis unspecified |
| N340 | Urethral abscess |
| N390 | Urinary tract infection site not specified |
| N410 | Acute prostatitis |
| N412 | Abscess of prostate |
| N431 | Infected hydrocele |
| N450 | Orchitis epididymitis and epididymoorchitis with abscess |
| N459 | Orchitis epididymitis and epididymoorchitis without abscess |
| N700 | Acute salpingitis and oophoritis |
| N709 | Salpingitis and oophoritis unspecified |
| N710 | Acute inflammatory disease of uterus |
| N719 | Inflammatory disease of uterus unspecified |
| N730 | Acute parametritis and pelvic cellulitis |
| N732 | Unspecified parametritis and pelvic cellulitis |
| N733 | Female acute pelvic peritonitis |
| N735 | Female pelvic peritonitis unspecified |
| N764 | Abscess of vulva |
| O030 | Spontaneous abortion incomplete complicated by genital tract and pelvic infection |
| O035 | Spontaneous abortion complete or unspecified complicated by genital tract and pelvic infection |
| O040 | Medical abortion incomplete complicated by genital tract and pelvic infection |
| O045 | Medical abortion complete or unspecified complicated by genital tract and pelvic infection |
| O050 | Other abortion incomplete complicated by genital tract and pelvic infection |
| O055 | Other abortion complete or unspecified complicated by genital tract and pelvic infection |
| O060 | Unspecified abortion incomplete complicated by genital tract and pelvic infection |
| O065 | Unspecified abortion complete or unspecified complicated by genital tract and pelvic infection |
| O070 | Failed medical abortion complicated by genital tract and pelvic infection |
| O075 | Other and unspecified failed attempted abortion complicated by genital tract and pelvic infection |
| O230 | Infections of kidney in pregnancy |
| O231 | Infections of bladder in pregnancy |
| O232 | Infections of urethra in pregnancy |
| O233 | Infections of other parts of urinary tract in pregnancy |
| O234 | Unspecified infection of urinary tract in pregnancy |
| O235 | Infections of the genital tract in pregnancy |
| O239 | Other and unspecified genitourinary tract infection in pregnancy |
| O411 | Infection of amniotic sac and membranes |
| O740 | Aspiration pneumonitis due to anaesthesia during labour and delivery |
| O753 | Other infection during labour |
| O85 | Puerperal sepsis |
| O860 | Infection of obstetric surgical wound |
| O861 | Other infection of genital tract following delivery |
| O862 | Urinary tract infection following delivery |
| O863 | Other genitourinary tract infections following delivery |
| O910 | Infection of nipple associated with childbirth |
| O911 | Abscess of breast associated with childbirth |
| O912 | Nonpurulent mastitis associated with childbirth |
| O981 | Syphilis complicating pregnancy childbirth and the puerperium |
| O982 | Gonorrhoea complicating pregnancy childbirth and the puerperium |
| O983 | Other infections with a predominantly sexual mode of transmission complicating pregnancy childbirth and the puerperium |
| O984 | Viral hepatitis complicating pregnancy childbirth and the puerperium |
| O985 | Other viral diseases complicating pregnancy childbirth and the puerperium |
| O986 | Protozoal diseases complicating pregnancy childbirth and the puerperium |
| O988 | Other maternal infectious and parasitic diseases complicating pregnancy childbirth and the puerperium |
| O989 | Unspecified maternal infectious or parasitic disease complicating pregnancy childbirth and the puerperium |
| P360 | Sepsis of newborn due to streptococcus group B |
| P361 | Sepsis of newborn due to other and unspecified streptococci |
| P362 | Sepsis of newborn due to Staphylococcus aureus |
| P363 | Sepsis of newborn due to other and unspecified staphylococci |
| P364 | Sepsis of newborn due to Escherichia coli |
| P365 | Sepsis of newborn due to anaerobes |
| P368 | Other bacterial sepsis of newborn |
| P369 | Bacterial sepsis of newborn unspecified |
| P38 | Omphalitis of newborn with or without mild haemorrhage |
| P398 | Other specified infections specific to the perinatal period |
| P769 | Intestinal obstruction of newborn unspecified |
| P77 | Necrotizing enterocolitis of fetus and newborn |
| R02 | Gangrene not elsewhere classified |
| T802 | Infections following infusion transfusion & therap inject |
| T814 | Infection following a procedure, not elsewhere classified |
| T826 | Infect and inflammatory reaction due to cardiac valve pros |
| T827 | Infect inflamm reac due other card vasc devs implant and graft |
| T835 | Infect inflam react due pros dev implt & graft urinary syst |
| T845 | Infect and inflammatory reaction due to internal joint pros |
| T846 | Infect and inflamm react due int fixation dev [any site] |
| T857 | Inf inflamm react due oth int prosth devs implants & grafts |
